# 

**Published:** 1859-03

**Authors:** 


### CONTENTS.

### ORIGINAL COMMUNICATIONS.

PAGE.

ARTICLE I.—An Essay—Read before the Atlanta Medical Society. By Hayden Coe, M. D. Atlanta, Ga., 389

ARTICLE II.—Cases from the Note Book of J. R. Cushing, M. D. of South Butler, Ala., 394

ARTICLE III.—Chloroform in Labor. By Jos. P. Logan, M. D. Atlanta, Georgia, 398

ARTICLE IV.—College Clinic. By J. G. Westmoreland, M. D. Atlanta, Georgia, 405

### SELECTIONS.

Remarks on some affections of the Spinal Column, 408
Milk Sickness: Its Etiology, Pathology, Diagnosis and Treatment, 414
Phosphorus in the Treatment of Phthisis, 417
The Pious Physician, or the Claims of Religion upon the Medical Profession, 419
Astonishing Impudence, 431
A Grain of Wheat from a Bushel of Chaff, 435

### EDITORIAL AND MISCELLANEOUS.

The Atlanta Medical College, 442

Extracts from Private Correspondence, 444

Mr. Eugene A. Groux—"Congenital Fissure of the Sternum," 4[illegible]6

Medical News and Items, 448

Quackery in France, 449

Scarcity of Women in Victoria, 450

University of the Pacific—Medical Department, 450

The Use of Snuff, 451

Errata, 451

Subscriptions, 451

Meteorological Table, 452

